# Supplementary material for: DNA origami-based single-molecule force spectroscopy elucidates RNA Polymerase III pre-initiation complex stability
Source: Nat Commun. 2020 Jun 5;11:2828. doi: 10.1038/s41467-020-16702-x (PMC7275037; doi:10.1038/s41467-020-16702-x)
Supplement: Supplementary file 1 — Supplementary Information [file 41467_2020_16702_MOESM1_ESM.pdf]

Supplementary Information

DNA origami-based single-molecule force spectroscopy elucidates RNA Polymerase III pre-initiation complex stability

Kramm et al.

Supplementary Figures

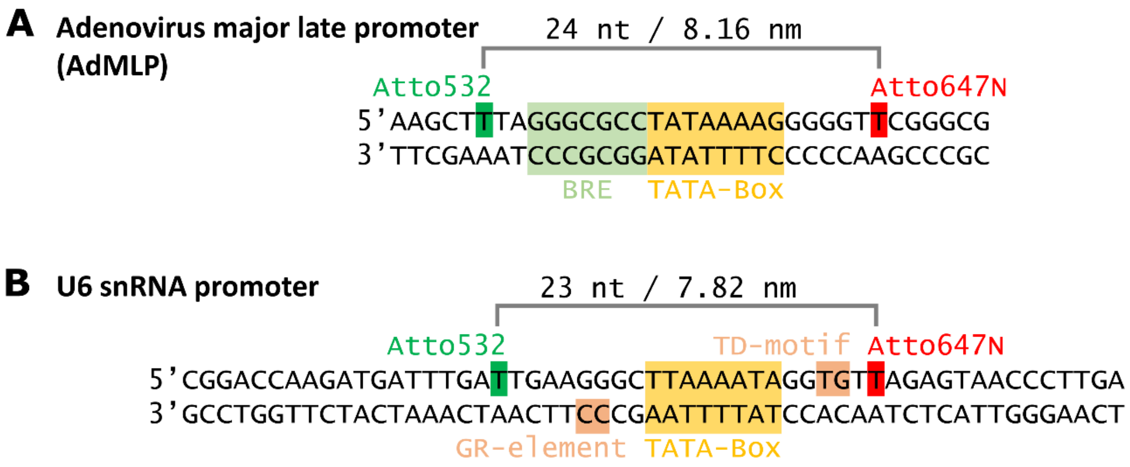

Supplementary figure 1: Schematic overview of the used promoter DNA sequences. A) DNA sequence of the Adenovirus major late promoter (AdMLP). The TATA-box element (yellow), bound by TBP, the B recognition element (BRE, light green) bound by TFIIB and the position of the donor (green) and acceptor fluorophore (red) are indicated by colour. **B)** DNA sequence of the U6 snRNA promoter. The TATA-box element (yellow), bound by TBP, the GR-element and TD-motif (orange) bound by Brf2 and the position of the donor (green) and acceptor fluorophore (red) are indicated by colour.

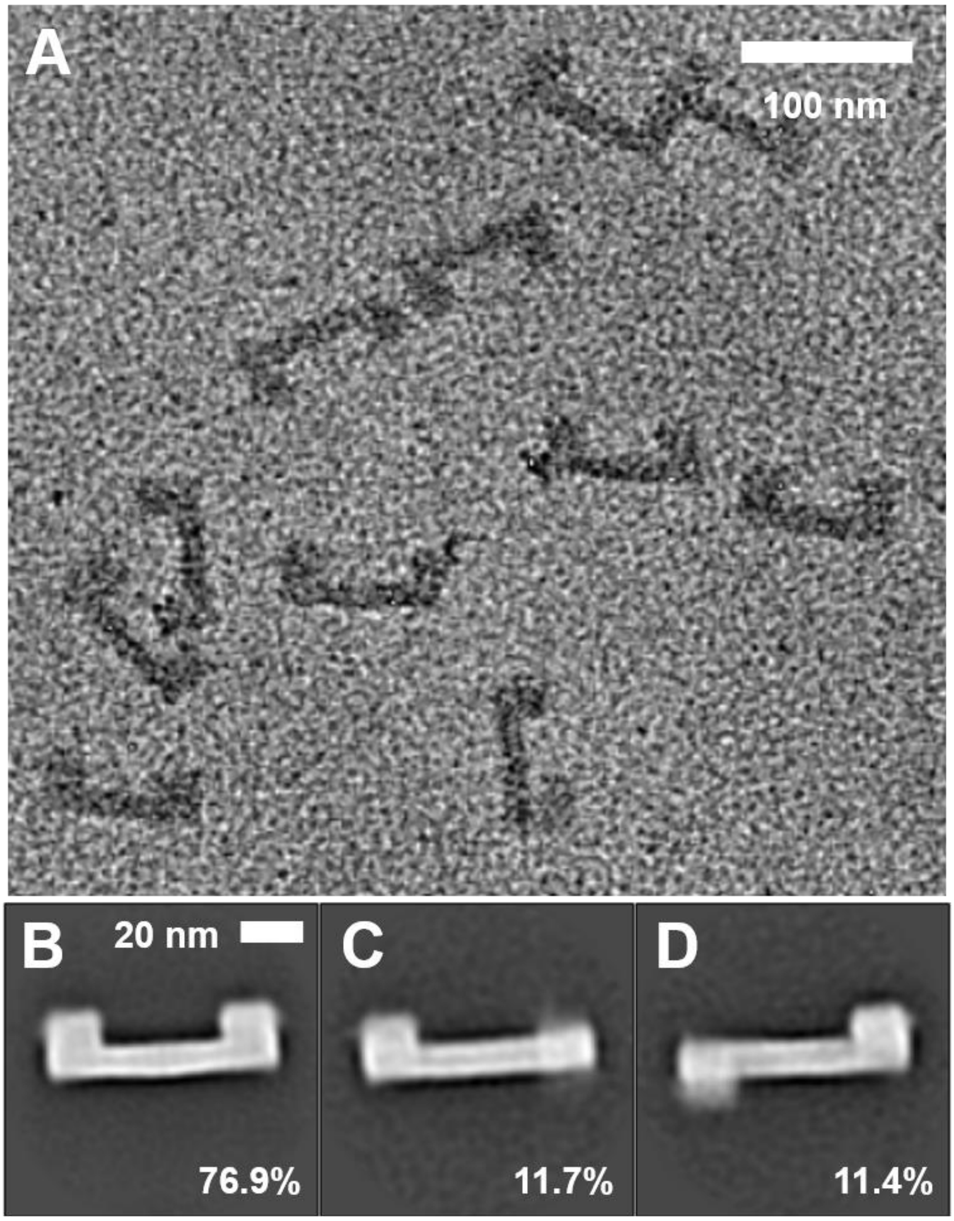

Supplementary Figure 2: Electron Microscopy characterization 3pN DNA origami force clamps. A) Exemplary section of electron micrograph showing unstained 'force clamps'. Scale bar: 100 nm. **B**) 2D-class average 1 (Methods) comprises 76.9% of particles and shows mainly intact origamis with limited flexibility in peripheral regions as previously observed [1]. Scale bar: 20 nm. **C**) 2D-class average 2 comprises 11.7% of particles. Unstained particles in this class appear to be damaged either during origami assembly or grid-preparation. Scale bar: 20 nm. **D**) 2D-class average 3 comprises 11.4% of particles. Particles in this class may be in top-view and/or could be damaged. Class averages were calculated from 2133 particles recorded in one experiment.

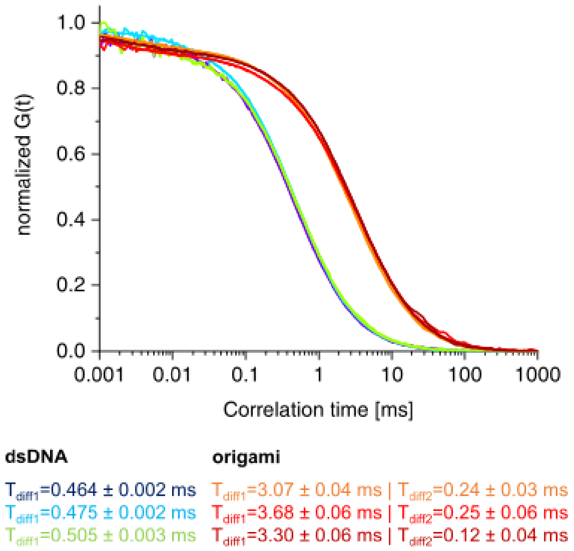

Supplementary Figure 3. Fluorescence correlation spectroscopy monitors diffusion behaviour of a DNA origami force clamp compared to the respective short double-stranded promoter DNA. The autocorrelation function G(t) of the acceptor signal of the short dsDNA U6 promoter (55 nt) and the U6 DNA origami force clamp were calculated. The decay was fitted with a one- (dsDNA) or two-component (DNA origami force clamp) fit function to calculate the relative diffusion time T_{diff} (given with standard error of the fit). Data for three independent experiments are shown.

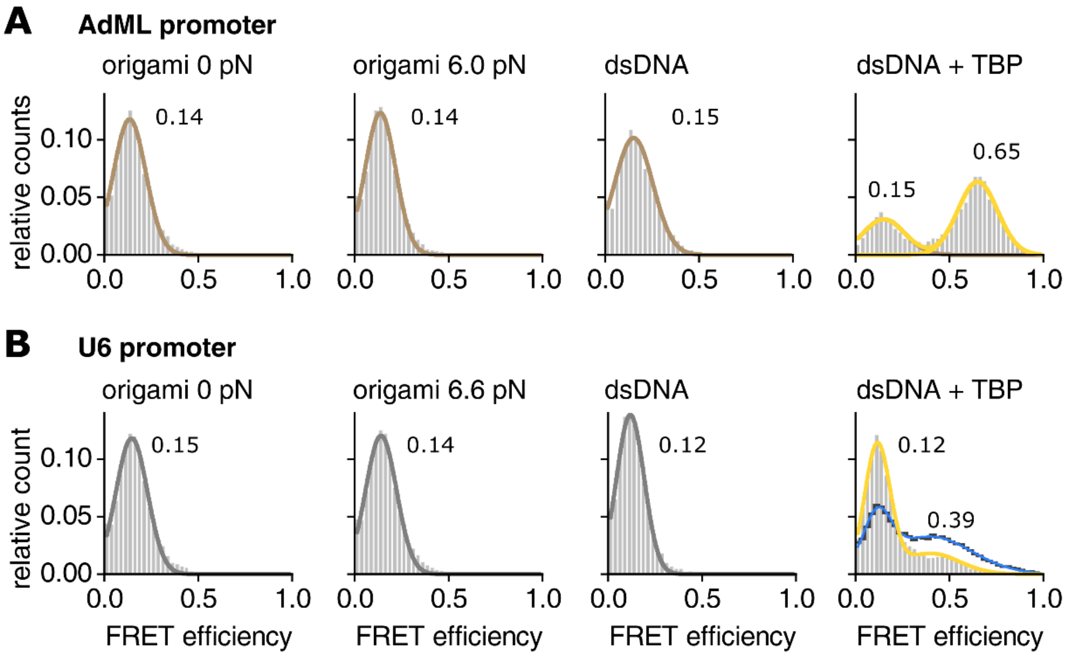

Supplementary Figure 4: Confocal single-molecule FRET experiments comparing FRET efficiencies of the DNA origami and linear double stranded DNA oligonucleotides. The exact same doubly labelled DNA oligonucleotide was used to form a double-stranded promoter DNA as part of the DNA origami or linear double stranded DNA to form the double-stranded **A**) AdMLP or **B**) U6 promoter. The proteins were used in the following concentrations: (A) TBP = 20, (B) TBP = 20 nM (histogram and yellow line for the Gaussian fit), TBP = 100 nM (blue line) – this measurement has been carried out to determine the FRET efficiency of the high FRET state on the U6 promoter (E=0.39) using linear dsDNA. DsDNA refers to the linear dsDNA without the DNA origami.

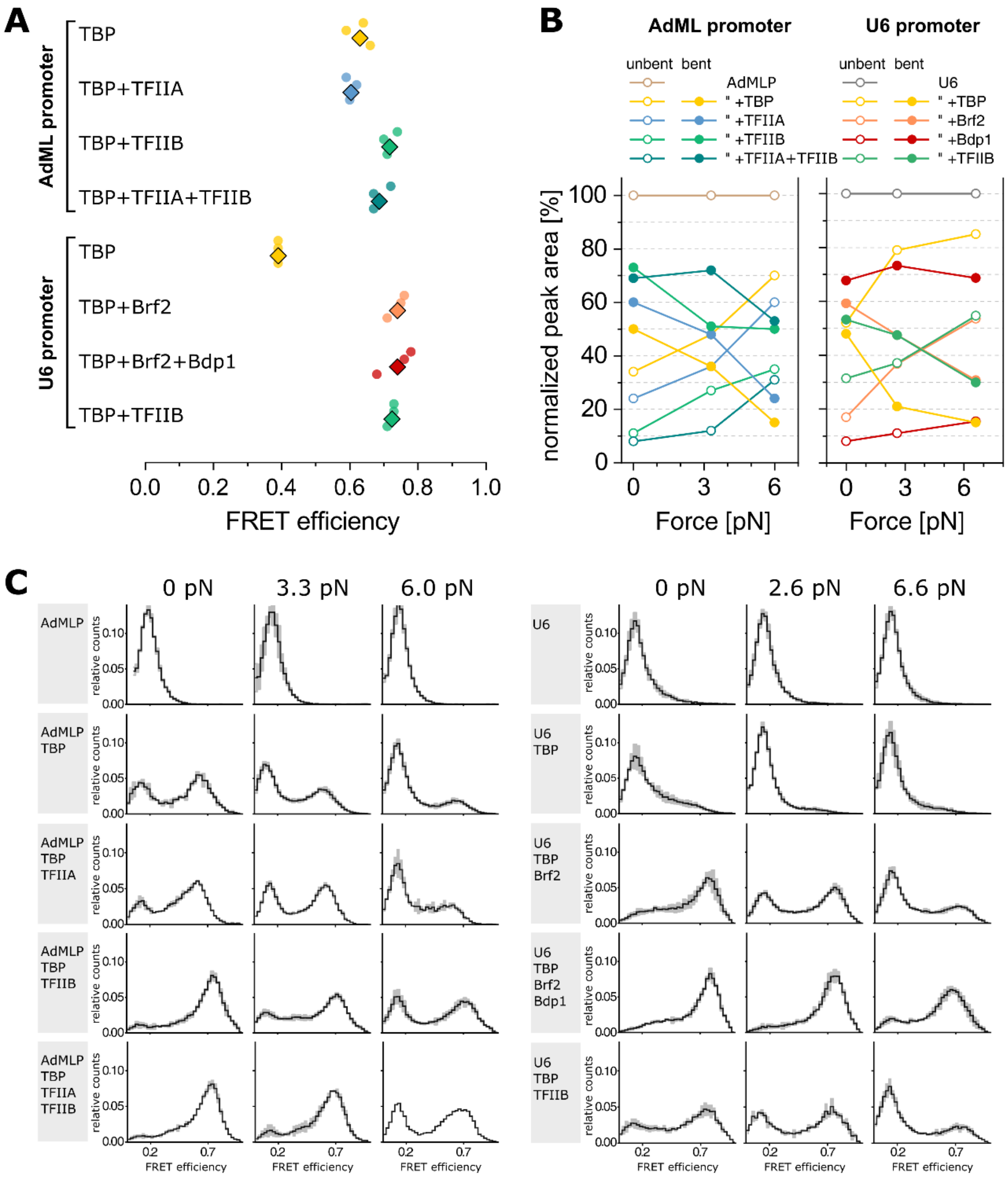

Supplementary Figure 5: Comparison of FRET efficiencies and peak areas of fitted FRET population for RNAP II and III initiation complexes. **A**) The mean FRET efficiency (diamonds) of the high FRET population for all three forces (0, 3.3/2.6, 6.0/6.6 pN, indicated by dots) is summarized. **B**) Changes in fitted peak area of the low FRET (unbent DNA) and high FRET (bent DNA) population under different forces. All areas were normalized to the total fitted area (see Supplementary Table 2). The plotted DNA/protein complexes include DNA+TBP (yellow), DNA+TBP+TFIIA (blue), DNA+TBP+TFIIB (green), DNA+TBP+TFIIA+TFIIB (teal), DNA+TBP+Brf2 (orange), DNA+TBP+Brf2+Bdp1 (red). **C**) Overview of all FRET efficiency histograms from confocal experiments. The mean FRET efficiency (black) and standard deviation of triplicates (grey) are shown. Protein concentrations used as follows: TFIIB (0.2 µM), TFIIA (2 µM), all other proteins (20 nM).

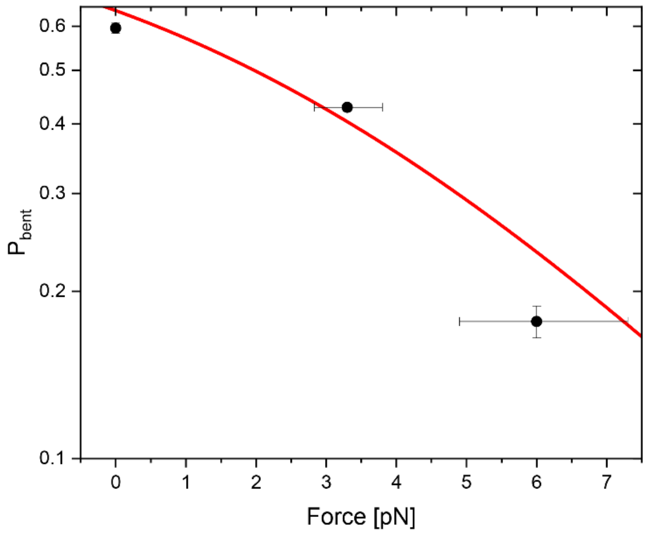

Supplementary Figure 6: **Calculation of bent state probability under force.** Logarithmic plot of the probability of the bent state P_{bent} for the TBP-AdMLP promotor complex as a function of force. The red solid line is the fit of the Boltzmann distribution P_{bent} yielding Δx of 1.2 ± 0.4 nm and ΔG of 2.3 ± 1.4 pN·nm. Mean and y-axis error of P_{bent} derived via error propagation of the standard error of Gaussian fit areas (Supplementary Note 2; $n_{0\ pN}$= 26187, $n_{3.3\ pN}$ = 48743 and $n_{6.0\ pN}$ = 37981).; the x-axis error is the uncertainty of the calculated forces resulting from the standard deviation of the single base length of 0. 63 ± 0.08 nm [1].

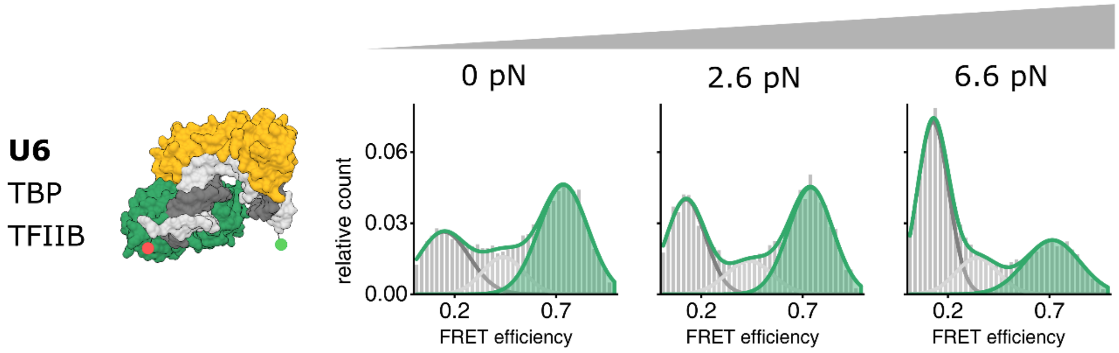

Supplementary Figure 7: Force dependency of promoter binding of RNAP II initiation factors at a canonical RNAP III promoter. A) Structural models (PDB: 1C9B) of the U6 snRNA promoter (U6, grey) in an unbent conformation and the bend state bound by TBP (yellow), and TFIIB (green). **B)** Single-molecule FRET measurements on diffusing molecules monitor TBP-induced DNA bending after addition of TBP (20 nM) and TFIIB (200 nM) to the U6 DNA origami force clamps at increasing forces (0, 2.6, 6.6 pN). FRET efficiency histograms showing the relative distribution between the unbent DNA state (low FRET population, E = 0.13, dark grey) and TBP-induced bent state (high FRET population, E = 0.73, green). Low and high FRET populations were fitted with a Gaussian distribution. Each measurement was carried out at least three times.

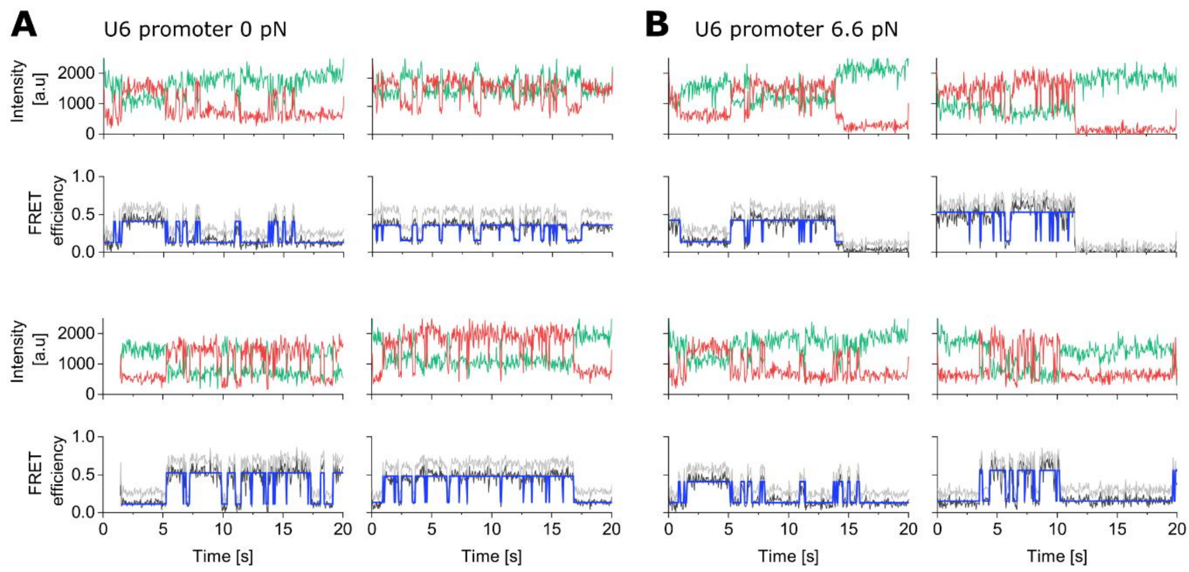

Supplementary Figure 8: Representative traces from single-molecule TIRF experiments following the interaction of HsTBP binding to a U6 promoter at A) 0 pN and 6.6 pN force. The donor emission after donor excitation (green), acceptor emission after donor excitation (red), proximity ratio (grey), FRET efficiency (black, corrected for cross talk, direct excitation and gamma) and fit with a two-state Hidden Markov model (blue) are shown.

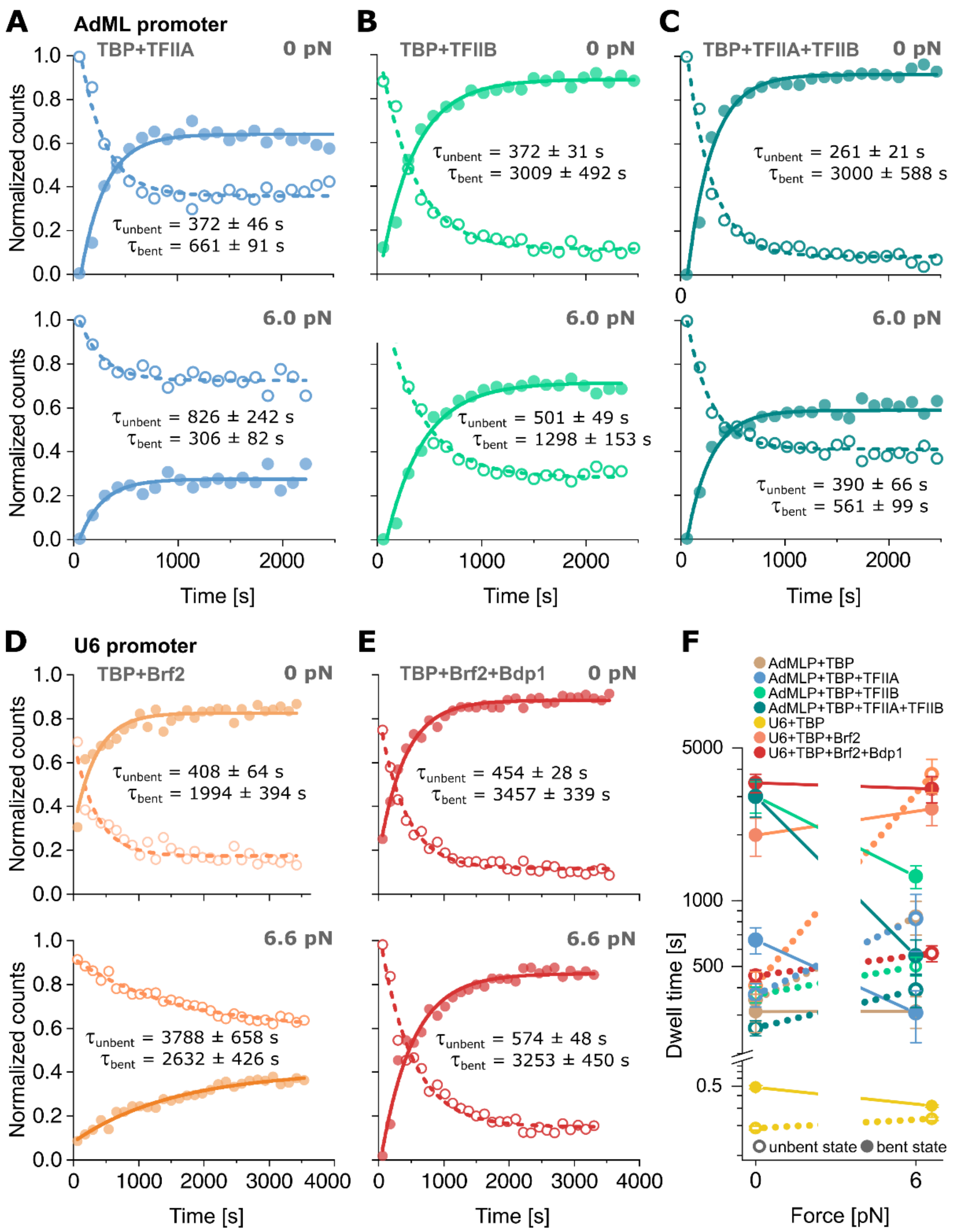

Supplementary Figure 9: Force dependency of TBP DNA-bending kinetics.

Relative ratios of low FRET (open circle) to high FRET state (filled circle) from a confocal kinetics experiment with **A)** TBP (20 nM) and TFIIA (2 µM), **B)** TBP (20 nM) and TFIIB (200 nM), C) TBP (20 nM), TFIIA (2 µM) and TFIIB (200 nM) **D)** TBP and Brf2 (20 nM each) and **E)** TBP, Brf2 and Bdp1 (20 nM each) binding to the AdML promoter at 0 pN and 6.0 pN or the U6 promoter force clamp at 0 pN and 6.0 pN force. Data were fitted with a mono-exponential function. Dwell times were calculated by deconvolution with a perturbation-relaxation model. **F)** Comparison of dwell times in the bent and unbent state for TBP-containing initiation complexes at 0 pN and 6.0 pN (AdMLP) or 6.6 pN (U6). Mean ± s.e.m. derived via error propagation from the exponential fit (see Supplementary Table 3 for the number of analysed molecules). Connecting lines between data points are meant as visual guides and do not represent interpolations.

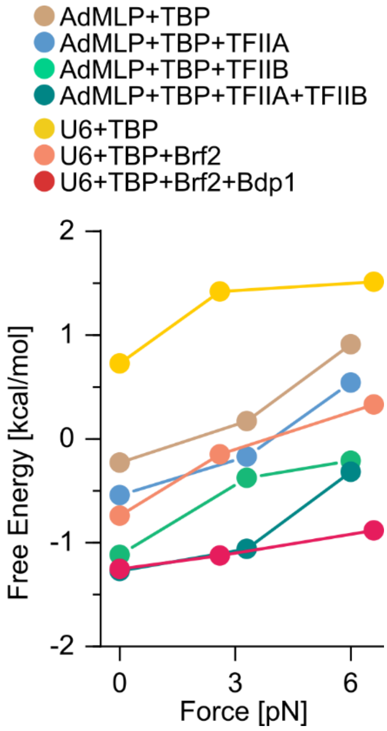

Supplementary Figure 10: Gibbs free energy of initiation factor complexes under force.

Complex free energies as a function of force were calculated based on the ratio of the bent state to unbent state (data derived from equilibrium experiments).

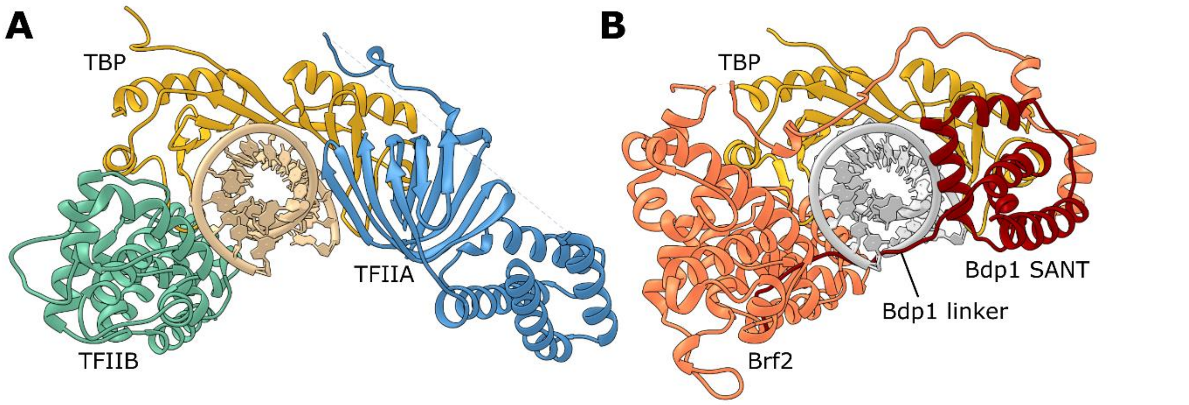

Supplementary Figure 11: Structural comparison of RNAP II and III initiation factor complexes.
Ribbon representation of the crystal structures of A) the human AdMLP/TBP/TFIIA/TFIIB complex (PDB: 5IYB) and B) the human U6/TBP/Brf2/Bdp1 complex (PDB: 5N9G). The structures are color-coded: AdMLP-DNA (tan), TBP (yellow), TFIIA (blue), TFIIB (green), U6-DNA (grey), Brf2 (orange), Bdp1 (SANT domain and linker domain, red).

Supplementary Tables

Supplementary Table 1: Sequences of DNA oligonucleotides. The coupling position of the Atto532 (green) and Atto647N (red) fluorophore, the TATA box sequence (blue) and the BamHI restriction site (yellow) are indicated by color. The 5'-phosphate (Pho-) modification was used for enzymatic ligation (see below).

Name	Sequences and modifications of DNA oligonucleotides (5'-3')	Supplier
AdMLP forward	Pho-gatccTATGACTGCTTCGCGCCCGAACCCCCTTTTATAGGCGCCCTAa	Biomers
AdMLP reverse	Pho-agcttTAGGGCGCCTATAAAAGGGGGTTCGGGCGCGAAGCAGTCATAg	Biomers
U6 forward	Pho-gatccTATGATCAAGGGTTACTCTAACACCTATTTTAAGCCCTTCAATCAAA TCATCTTGGTCCGa	Biomers
U6 reverse	Pho-agcttCGGACCAAGATGATTTGATTGAAGGGCTTAAAATAGGTGTTAGAGTA ACCCTTGATCATAg	Biomers
MLP_D	AAGCTTTAGGGCGCCTATAAAAG	IBA
MLP_A	Pho-GGGGTTCGGGCG	IBA
MLP_lig	TTCGCGCCCGAACCCCCTTTTATAGGCGC	Sigma
MLP_TS	CGCCCGAACCCCCTTTTATAGGCGCCCTAAAGCTT	MWG
U6_D	CGGACCAAGATGATTTGATTGAAGGGCTTAAAATA	IBA
U6_A	Pho-GGTGTTAGAGTAACCCTTGA	IBA
U6_Lig	GGTTACTCTAACACCTATTTTAAGCCCTTC	Sigma
U6_TS	TCAAGGGTTACTCTCACACCTATTTTAAGCCCTTCAATCAAATCATCTTGGTCCG	Sigma
BamHI_comp	TCATAGGATCCCCGGGTA	Metabion
MLP_comp	AAGCTTTAGGGCGCCTATAAAAGGGGGTTCGGGCG	see below
U6_comp	CGGACCAAGATGATTTGATTGAAGGGCTTAAAATAGGTGTTAGAGTAACCCTTGA	see below
AdMLP 6pN_1	TTTTGCTTTCATCAACATTAAATCCGTAATCGTAACCTTGGGTACAGG	MWG
AdMLP 6pN_2	TTTTAAGTTTCATTCCTCTGGAGAGGCTATACGCCAGGGTTTTCCCAGT	MWG
AdMLP 6pN_3	TTTTCGCTCATGGACGAGCCG	MWG
AdMLP 6pN_4	TTTTACTTGCCTGAGTAGAAGTAATTCCACGATT	MWG
AdMLP 6pN_5	AGCCTTCACCGCCTGGCGTTATCCGCTCACATAAC	MWG
AdMLP 6pN_6	GCTACAACATAAATACCATTGCAACAGGAAAAATTTT	MWG
AdMLP 6pN_7	ATGCAATGGTGAGAAAGGCATGATTAAGGTGCATCAGATTGTAATT	MWG
AdMLP 6pN_8	GTCAATAGCAAGGCACAGGCACCTCAGAGCTTTAA	MWG
AdMLP 6pN_9	TTTCCTGTGTGAAATTCCTGAGAGGGGTCG	MWG
AdMLP 6pN_10	GGGGATGTGCTACCTGTTTAGCTTTTT	MWG

Supplementary Table 2: Average FRET efficiencies and peak areas of fitted FRET population for RNAP II and III initiation complexes. FRET efficiency histograms of three technical replicates were fitted with a single (DNA) or triple Gaussian distribution. The average FRET efficiency was determined as the centre of each fit population. The area of each peak was normalized to the total fit area. *The value was fixed for fitting. **Peak 2 was included to allow more accurate fitting of the low and high FRET population with a fixed area of 0.004 or *** 0.006.

	# Molecules	Average FRET efficiency			Normalized peak area [%]			R^2
		Peak 1 (unbent DNA)	Peak 2**	Peak 3 (bent DNA)	Peak 1 (unbent DNA)	Peak 2**	Peak 3 (bent DNA)	
**AdML promoter**								
0 pN DNA	27729	0.14			100			0.99
0 pN TBP	26187	0.12	0.38	0.64	34	16	50	0.98
0 pN TBP+TFIIA	33217	0.13	0.37	0.59	26	16	59	0.99
0 pN TBP+TFIIB	36027	*0.12	0.47	0.74	11	16	73	0.99
0 pN TBP+TFIIA+TFIIB	37846	*0.13	0.47	0.72	7	***24	69	0.99
3.3 pN DNA	40289	0.15			100			0.99
3.3 pN TBP	48743	0.10	0.31	0.59	48	15	36	0.99
3.3 pN TBP+TFIIA	27514	0.13	0.36	0.62	36	16	48	0.99
3.3 pN TBP+TFIIB	59994	0.11	0.40	0.70	26	***23	51	0.99
3.3 pN TBP+TFIIA+TFIIB	29419	0.13	0.38	0.67	12	16	72	0.99
6 pN DNA	33930	0.14			100			0.99
6 pN TBP	37981	0.13	*0.40	0.66	70	16	15	0.99
6 pN TBP+TFIIA	24865	0.13	0.36	*0.60	58	16	26	0.99
6 pN TBP+TFIIB	35307	0.13	0.40	0.71	35	16	49	0.99
6 pN TBP+TFIIA+TFIIB	20817	0.13	0.38	0.67	31	16	53	0.98
**U6 snRNA promoter**								
0 pN DNA	25170	0.15			100			0.96
0 pN TBP	31961	0.14		*0.39	52		48	0.99
0 pNTBP+Brf2	33693	*0.16	0.45	0.76	17	***24	60	0.97
0 pN_TBP+Brf2+Bdp1	30453	*0.16	0.47	0.78	8	***24	68	0.98
2.6 pN DNA	25960	0.15			100			0.98
2.6 pN TBP	26820	0.14		*0.39	79		21	0.99
2.6 pN TBP+Brf2	44296	0.15	0.45	0.75	37	16	47	0.98
2.6 N TBP+Brf2+Bdp1	25724	*0.16	0.50	0.76	11	16	73	0.99
6.6 pN DNA	49120	0.15			100			0.99
6.6 pN TBP	10178	0.13		*0.39	85		15	0.99
6.6 pN TBP+Brf2	45829	0.15	0.38	0.71	54	16	31	0.99
6.6 pN TBP+Brf2+Bdp1	48274	*0.15	0.39	0.68	16	16	69	0.99
0 pN TBP+TFIIB	21659	0.15	0.43	0.73	31	15	53	0.96
2.6 pN TBP+TFIIB	18543	0.12	0.44	0.73	37	15	48	0.96
6.6 pN TBP+TFIIB	21223	0.13	0.34	0.72	55	16	30	0.99

Supplementary Table 3: Fit values for determined for kinetics measurements. Perturbation-relaxation time course experiments of three technical replicates were combined and the decay of the low FRET population was fitted with a mono-exponential decay function to determine the decay constant and the relative ratio of the low FRET population in dynamic equilibrium, y0. Values given are mean ± s.e.m.

Complex	Force [pN]	# Molecules	Fit decay constant [s]	Fit y0	R^2 of the fit
AdMLP + TBP	0	56851	165 ± 29	0.53 ± 0.01	0.93
“	6.0	64111	228 ± 36	0.73 ± 0.01	0.87
AdMLP + TBP + TFIIA	0	40563	238 ± 26	0.36 ± 0.01	0.95
“	6.0	32081	223 ± 57	0.73 ± 0.01	0.81
AdMLP + TBP + TFIIB	0	17570	331 ± 24	0.11 ± 0.01	0.98
“	6.0	67574	361 ± 30	0.28 ± 0.01	0.98
AdMLP + TBP + TFIIA + TFIIB	0	50561	240 ± 17	0.08 ± 0.01	0.98
“	6.0	22599	230 ± 35	0.41 ± 0.01	0.88
U6 + TBP + Brf2	0	23607	339 ± 49	0.17 ± 0.01	0.89
“	6.6	63193	1553 ± 209	0.59 ± 0.02	0.97
U6 + TBP + Brf2 + Bdp1	0	44005	401 ± 22	0.12 ± 0.01	0.98
“	6.6	61715	488 ± 36	0.15 ± 0.01	0.97

Supplementary Notes

Supplementary Note 1: Calculation of forces for the AdML promoter and U6 promoter DNA origami force clamps

Forces were calculated according to reference [1] where the ssDNA is described as a freely-jointed chain (FJC). The width of the double-stranded promotor is the average value of the bent and unbent state. For the AdML promotor we calculated an average width of 10.17 nm and for the U6 promotor of 16.01 nm. For new scaffold and promotor length the forces for the 2.5 pN staple set from reference [1] are calculated to be 3.3 pN for the AdML promotor and 2.6 pN for the U6 promotor. The 6.2 pN staple set of reference [1] results in a 6.6 pN force for the U6 promotor. The 6.0 pN for the AdML promotor were newly designed. Staples were ordered from Eurofins MWG and are listed in Supplementary Table 1.

Supplementary Note 2: TBP induced bending probability

We calculate the force dependent bending probability P_{bent} from the high and low FRET areas of the Gaussian fits.

$$P_{\text{bent}} = \frac{\text{area}_{\text{high FRET}}}{\text{area}_{\text{high FRET}} + \text{area}_{\text{low FRET}}} \quad (1)$$

P_{bent} is related to the Boltzmann distribution by the change in free energy ΔG:

$$P_{\text{bent}} = \frac{\exp\left(\frac{\Delta G - F \cdot \Delta x}{\text{k}_{\text{B}} T}\right)}{1 + \exp\left(\frac{\Delta G - F \cdot \Delta x}{\text{k}_{\text{B}} T}\right)} \quad (2)$$

where ΔG is the free energy between the undistorted state and bent state at 0 pN. F is the applied force to the promotor DNA and Δx is the distance change along the one-dimensional reaction coordinate (Supplementary Figure 6).

Supplementary References

1. Nickels, P. C. *et al.* Molecular force spectroscopy with a DNA origami-based nanoscopic force clamp. *Science (New York, N.Y.)* **354,** 305–307; 10.1126/science.aah5974 (2016).
